# High Viral Diversity and Mixed Infections in Cerebral Spinal Fluid From Cases of Varicella Zoster Virus Encephalitis

**DOI:** 10.1093/infdis/jiy358

**Published:** 2018-07-07

**Authors:** Daniel P Depledge, Juliana Cudini, Samit Kundu, Claire Atkinson, Julianne R Brown, Tanzina Haque, Charlotte J Houldcroft, Evelyn S Koay, Fiona McGill, Richard Milne, Tom Whitfield, Julian W Tang, Gillian Underhill, Tomas Bergstrom, Peter Norberg, Richard Goldstein, Tom Solomon, Judith Breuer

**Affiliations:** 1 Division of Infection and Immunity, University College London; 2 School of Human and Life Sciences, Canterbury Christ Church University, University of Kent; 3 Department of Virology, Royal Free Hospital, London; 4 Microbiology, Virology and Infection Prevention and Control, Great Ormond Street Hospital NHS Foundation Trust, London; 5 Institute of Infection and Global Health, University of Liverpool; 6 National Institute for Health Research, Health Protection Research Unit in Emerging and Zoonotic Infections, University of Liverpool; 7 Royal Liverpool University Hospitals; 8 Clinical Microbiology, University Hospitals of Leicester NHS Trust; 9 Departments of Clinical Microbiology, Pathology Centre, Queen Alexandra Hospital, Portsmouth; 10 Walton Centre NHS Foundation Trust, Liverpool, United Kingdom; 11 Department of Pathology, National University of Singapore; 12 Molecular Diagnosis Centre, National University Hospital, Singapore; 13 Department of Infectious Diseases, Section for Clinical Virology, Institute of Biomedicine, University of Gothenburg, Sweden

**Keywords:** varicella zoster virus, encephalitis, viral population, evolution, neurotropism, pathogenesis

## Abstract

**Background:**

Varicella zoster virus (VZV) may cause encephalitis, both with and without rash. Here we investigate whether viruses recovered from the central nervous system (CNS; encephalitis or meningitis) differ genetically from those recovered from non-CNS samples.

**Methods:**

Enrichment-based deep sequencing of 45 VZV genomes from cerebral spinal fluid (CSF), plasma, bronchoalveolar lavage (BAL), and vesicles was carried out with samples collected from 34 patients with and without VZV infection of the CNS.

**Results:**

Viral sequences from multiple sites in the same patient were identical at the consensus level. Virus from vesicle fluid and CSF in cases of meningitis showed low-level diversity. By contrast, plasma, BAL, and encephalitis had higher numbers of variant alleles. Two CSF-encephalitis samples had high genetic diversity, with variant frequency patterns typical of mixed infections with different clades.

**Conclusions:**

Low viral genetic diversity in vesicle fluid is compatible with previous observations that VZV skin lesions arise from single or low numbers of virions. A similar result was observed in VZV from cases of VZV meningitis, a generally self-limiting infection. CSF from cases of encephalitis had higher diversity with evidence for mixed clade infections in 2 cases. We hypothesize that reactivation from multiple neurons may contribute to the pathogenesis of VZV encephalitis.

## Teaser

Cerebrospinal fluid varicella zoster virus (VZV) from encephalitis (8) was significantly more diverse than vesicular fluid (24) (*P* < .05) with mixed clade infection in 2. Nonvaricella-associated VZV encephalitides may be caused by simultaneous viral reactivation from multiple neurons.

Primary infection with varicella zoster virus (VZV), an alphaherpesvirus, causes chickenpox (varicella). Following latency in the neurons of peripheral sensory ganglia, VZV reactivates in 30% to cause herpes zoster (shingles). Around 50% of herpes zoster cases occur in subjects with reduced cell-mediated immunity [1–3]. In common with other alphaherpesviruses, VZV is able to infect the central nervous system (CNS) to cause encephalitis, either as a complication of varicella or herpes zoster, but also in the absence of rash.

Studies of equid herpesvirus1 (an alphaherpesvirus of horses) have reported that a naturally occurring point mutation in the viral DNA polymerase significantly increases the risk of encephalitis [4]. Here we examine whether VZV that infects the CNS shares certain sequence motifs either uniquely or more frequently than non-CNS VZV infections. VZV exclusively infects humans and there are no animal models that recapitulate the biology or clinical presentation of VZV encephalitis.

## METHODS

### Sample Collection and Ethics

The samples (Table 1) used in this study were obtained either as part of routine care or through prospective research/clinical studies. Four cerebrospinal fluid (CSF) samples were obtained via the UK Meningitis Study, where ethical approval was given by the North Wales Multicentre Research Ethics Committee (reference 11/WA/0218). All samples were subsequently transferred to Great Ormond Street Hospital for Children (GOSH) for processing. Purified DNA was stored at −80°C prior to sequencing. The use of all samples for research was subsequently approved through the University College London Partners Infection DNA Bank by the National Research Ethics Service Committee, London, Fulham (Research Ethics Committee reference 12/LO/1089). All samples tested negative for the VZV live-attenuated vaccine.

**Table 1.** Details of Patient Samples Sequenced in This Study

Identity	Age	Source Country	Sample Type	Immune Status	Note	Diversity	Number of Variant Alleles
VES1	55 (Patient A)	UK	Vesicle	Immunocompromised	Varicella	4.20E-05	2
BAL1			BAL			1.58E-04	33
PLAS1			Plasma			4.90E-05	5
VES2	67 (Patient B)	UK	Vesicle	Immunocompromised	Zoster (disseminated)	3.97E-05	8
SPU			Sputum			3.28E-05	6
PLAS2			Plasma			8.02E-04	103
CSF3	76 (Patient C)	UK	CSF	Immunocompetent	Encephalitis with Zoster	9.75E-05	23
VES27			Vesicle			4.39E-05	1
VES18	2 (Patient D)	UK	Vesicle	Immunocompetent	Varicella	7.75E-08	9
VES19			Vesicle			1.60E-05	17
VES20			Vesicle			4.83E-06	2
VES21			Vesicle			3.34E-05	2
VES22			Vesicle			2.58E-05	0
VES4	8 (Patient E)	UK	Vesicle	Immunocompetent	Varicella	3.70E-05	2
VES26						3.37E-05	0
VES6	3 (Patient F)	UK	Vesicle	Immunocompetent	Varicella	5.81E-06	25
VES25						5.14E-05	0
PLAS3	8	UK	Plasma	Immunocompromised	Zoster	2.83E-06	16
BAL2	10	UK	BAL	Immunocompetent	Zoster	4.45E-05	25
CSF1	42	UK	CSF	Immunocompetent	Encephalitis	4.58E-04	133
CSF2	32	UK	CSF	Immunocompetent	Encephalitis	2.38E-04	169
CSF4	81	Sweden	CSF	Immunocompetent	Encephalitis	3.42E-05	21
CSF5	70	Sweden	CSF	Immunocompetent	Encephalitis	6.24E-05	31
CSF6	58	Sweden	CSF	Immunocompetent	Encephalitis	8.35E-05	14
CSF7	54	Singapore	CSF	Immunocompetent	Encephalitis + Zoster	3.30E-05	85
CSF8 (UKM1018^a^)	28	UK	CSF	Immunocompetent	Meningitis	2.81E-05	11
CSF9 (UKM1211^a^)	29	UK	CSF	Immunocompromised	Meningitis	6.03E-05	5
CSF10 (UKM0338^a^)	22	UK	CSF	Immunocompetent	Meningitis	2.23E-06	6
CSF11 (UKM0624^a^)	69	UK	CSF	Immunocompetent	Meningitis	3.41E-05	2
CSF12	74	UK	CSF	Immunocompetent	Encephalitis	1.00E-04	0
VES3	61	UK	Vesicle	Immunocompromised	Zoster	5.64E-05	8
VES5	8	UK	Vesicle	Immunocompromised	Varicella	3.17E-05	1
VES7	62	Sweden	Vesicle	Immunocompetent	Zoster	1.34E-05	2
VES8	74	Sweden	Vesicle	Immunocompetent	Zoster	2.21E-05	3
VES9	69	Sweden	Vesicle	Immunocompetent	Zoster	1.52E-05	42
VES10	89	Sweden	Vesicle	Immunocompetent	Zoster	3.31E-05	6
VES11	76	Sweden	Vesicle	Immunocompromised	Zoster	2.43E-05	4
VES12	90	Sweden	Vesicle	Immunocompetent	Zoster	2.27E-05	2
VES13	80	Sweden	Vesicle	Immunocompetent	Zoster	4.79E-05	3
VES14	79	Sweden	Vesicle	Immunocompetent	Zoster	1.53E-05	3
VES15	83	Sweden	Vesicle	Immunocompetent	Zoster	4.70E-05	4
VES16	44	Singapore	Vesicle	Immunocompetent	Varicella	1.96E-05	3
VES17	4	Germany	Vesicle	Immunocompetent	Varicella	4.14E-05	6
VES23	10	Italy	Vesicle	Immunocompetent	Varicella	2.00E-05	0
VES24	66	Sweden	Vesicle	Immunocompetent	Zoster	1.22E-05	0

Abbreviations: BAL, bronchoalveolar lavage; CSF, cerebrospinal fluid; PLAS, plasma; SPU, sputum; VES, vesicle.

^a^UK Meningitis Study.

### Library Preparation, Deep Sequencing, and Assembly

Sequence libraries were constructed from 200 ng of starting DNA, derived from original GOSH DNA extracts, supplemented with human DNA (Promega, G1471) where necessary. DNA was sheared on a Covaris E220 (peak incident power, 175; duty factor, 5; cycle per burst, 200; treatment time = 150 s). Multiplexed libraries were run on Illumina MiSeq or NextSeq sequencers. Paired-end sequence data were demultiplexed and sequence reads trimmed to remove low quality 3′ bases and adapter sequences using TrimGalore (http://www.bioinformatics.babraham.ac.uk/projects/trim_galore/). Read pairs were aligned against the VZV reference strain Dumas (NC_001348) using BBMap (http://sourceforge.net/projects/bbmap/) while duplicate read pairs were removed using MarkDuplicates (http://broadinstitute.github.io/picard) and local realignment performed using IndelRealigner [5]. SAM, BAM, and mpileup files were parsed using SAMTools [6], VarScan2 [7], and custom PERL scripts to produce both a consensus sequence for each sample and frequency profile for sites with 2 or more alleles present. For further analysis variant alleles were filtered using bam-readcount (https://github.com/genome/bam-readcount) and the fpfilter function in VarScan2 [7]. These combine to flag and remove artefactual variant alleles generated by sequencing/mapping errors.

### Multiple Sequence Alignment and Network Phylogeny

Using Mafft v7 [8], consensus sequences were aligned to reference VZV sequences derived from the 6 major clades [9] (Supplementary Table 1) and visualized using SplitsTree4 [10]. The 5 small repeat regions, R1-R5, and the terminal repeat region were masked from the alignment prior to tree construction.

### Nucleotide Diversity Estimates

Base counts at each position of each sample’s read alignment were extracted to calculate within-host nucleotide diversity (π), defined as the average number of nucleotide differences between reads at a site [11] . Strand-bias and random error rates were estimated and corrected for using maximum likelihood methods.

### Statistical Analyses

The Student *t* test was used to compare numbers of variant alleles present in samples grouped according to pathology, sample type, and immune status. The Kolmogorov-Smirnov (nonparametric) test was used to discern whether variant allele frequency distributions differed between vesicle and nonvesicle populations.

## RESULTS

### Description of Samples and Sequencing

Forty-five diagnostic clinical samples, obtained from a total of 34 patients with VZV-related conditions, were sequenced following enrichment for VZV DNA [12, 13]. For most patients, a single sample was available from skin vesicles (VES), CSF, plasma (PLAS), sputum (SPU), or bronchoalveolar lavage (BAL) (Table 1). Six patients had samples from multiple compartments (Table 1), enabling intrahost comparisons of VZV populations. Reference-guided alignments of paired-end reads to the VZV reference strain Dumas (NC_001348) were generated for each sample. The percentage of VZV mapping reads (on-target reads), which we have previously shown can act as a proxy for pathogen load [14], ranged from 3.68% to 91.8% (median 79.7%). Genome coverage ranged from 98.46%–100% at 1 × coverage to 0.34%–100% at 20 ×, with 35/45 samples having over 75% coverage at 100 ×, reflecting the highly variable numbers of VZV-mapping reads in each dataset (Supplementary Table 2).

### VZV Consensus Sequences Do Not Differ Between Body Compartments

**Figure 1.**
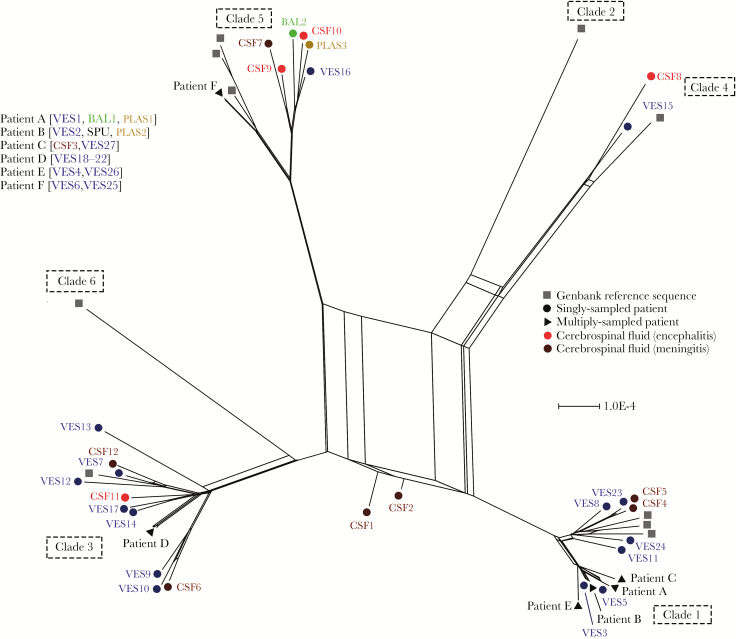
Network phylogeny identifies 2 putative interclade recombinant varicella zoster virus (VZV) genomes. Phylogenetic network of 55 VZV genomes, including 45 from this study and 10 representative the 6 major geographic clades (numbered 1–6). CSF1 and CSF2 represent putative interclade recombinants. Sample types are color coded. Abbreviations: BAL, bronchoalveolar lavage; CSF, cerebrospinal fluid; PLAS, plasma; SPU, sputum; VES, vesicle.

A consensus sequence was generated for each sample and aligned with 12 VZV genomes, representative of 6 different clades obtained from Genbank (Supplementary Table 1). A phylogenetic network [10] showed all but 2 sequences (CSF1 and CSF2) to cluster within established geographic clades (Figure 1). As described previously [9], the most parsimonious explanation at the time for the positioning of CSF1 and CSF2 was that both represent interclade recombinant strains. In 6 patients where multiple samples were obtained, VZV consensus sequences obtained from distinct compartments were identical. No VZV consensus genome sequences clustered by body compartment, and no single-nucleotide polymorphisms or sequence motifs were uniquely shared by VZV genomes present in the CSF (Figure 1).

### VZV Population Heterogeneity Varies by Body Compartment

**Figure 2.**
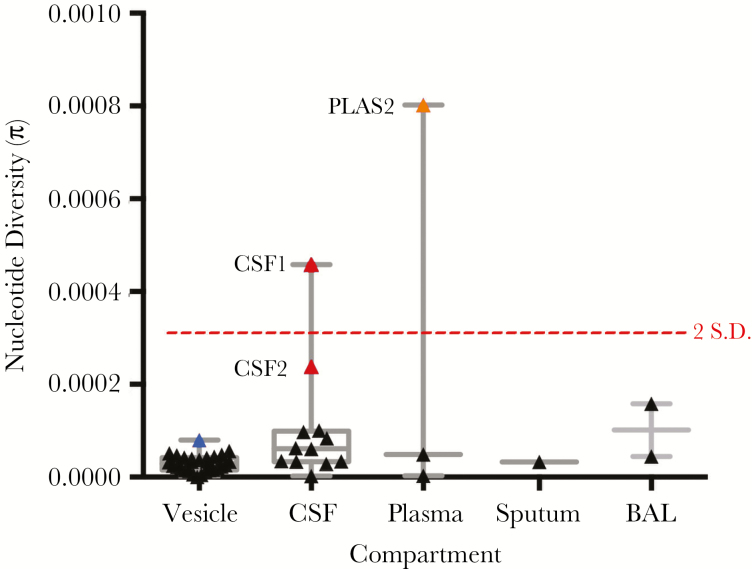
Intrahost nucleotide diversity (π) of varicella zoster virus (VZV) across compartments. Nucleotide diversity estimates of the VZV population in each individual sample is shown, grouped by sampled compartment. Median, interquartile range, and maximum-minimum range are marked, as is a dotted red line denoting nucleotide diversities that are 2 SD from the mean (calculated from the nucleotide diversities of all samples). Outlier CSF (red) and plasma (orange) samples are shown. The blue symbol represents the sum diversity of individual vesicles from patient D. Abbreviations: BAL, bronchoalveolar lavage; CSF, cerebrospinal fluid; PLAS, plasma.

We next generated estimates of within (intra-) sample nucleotide (population) diversity (Figure 2). Our method counts the numbers of reads supporting each allele at every site in the genome, calculates the nucleotide diversity at each position, and assigns a probability of the observed read distributions being true. The result computes diversity as a function of allele number and frequency, with larger numbers of low-frequency alleles generally having low diversity values. Nucleotide diversity (π) was lowest in vesicle fluid (mean π = 2.80E-05) compared to all other sampled compartments, including sputum (single sample, π = 3.28E-05), BAL (mean π = 1.01E-04), CSF (mean π = 1.03E-04), and plasma (median π = 2.85E-04). Nucleotide diversities of 2 samples, CSF1 and PLAS2, exceeded 2 standard deviations from the mean and were considered outliers (Figure 2). A second CSF sample, CSF2, while not statistically an outlier, was more diverse than the remaining samples (Figure 2).

**Figure 3.**
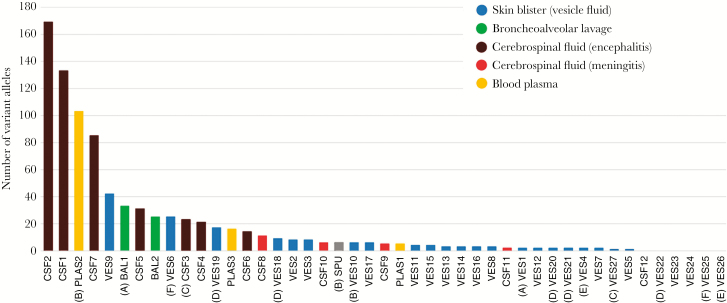
Total counts of filtered variant alleles per sample sequenced in this study. Sample identities correspond to samples shown in Table 1. Multiple-sampled patient are identified in parentheses (A–F). Abbreviations: BAL, bronchoalveolar lavage; CSF, cerebrospinal fluid; PLAS, plasma; SPU, sputum; VES, vesicle.

For subsequent analyses, variant alleles were filtered to flag and remove artefactual variant alleles generated by sequencing/mapping errors. Plots of the frequencies of filtered variant alleles and their positions along the genome are shown for vesicle (Supplementary Figure 1) and nonvesicle samples (Supplementary Figure 2). A second, vertical plot is included for each sample (shown to the right of the variant allele distribution plots) and shows the distribution of allele frequencies binned at 1% intervals (0%–100%) present in each sample irrespective of their genomic location (Supplementary Figures 1 and 2). Together, these plots were used to look for unusual patterns of variant allele frequency distributions. The VZV populations in each sample contained between 0 and 169 variant alleles (median = 6; Figure 3). Vesicle fluid sequences had the lowest numbers of variant alleles, that is between 0 and 42 (Figure 3). For patients A, B, and C, from whom multiple samples from different body compartments had been obtained, the vesicular fluid variants tended to be fewer than those present in other samples (Table 1 and Figure 3). Although individual vesicles sampled from the same individual (ie, patients D, E, and F), varied in their numbers of variants (ie, from 0 to17 for patient D, 0 to 22 for patient F, 0 and 4 for patient E; Table 1), in each case the population of variant alleles was distinct (Supplementary Figures 2 and 3). The majority of variant alleles in vesicles were present at low frequencies (95% < 20% frequency), regardless of total numbers, resulting in a distribution shifted towards low frequencies. For example although the numbers of variant alleles in VES6 and VES9 were the highest of vesicular fluid samples (25 and 42, respectively) the frequency with which they occurred was still very low (Supplementary Figure 2, vertical graphs). Similarly, for patients D, E, and F, although different vesicles had different numbers of alleles, the variant frequency profile was also low (Figure 3 and Supplementary Figure 2). While the vesicular and meningitis samples (CSF8, CSF9, CSF11, CSF12) largely had fewer than 10 variant alleles (24/27 samples and 3/4 samples, respectively) (Table 1 and Figure 3), 14/16 nonvesicular samples had more than 10 variant alleles (median = 19; Figure 3) with more evidence of high-frequency variants (Supplementary Figure 1). The difference between allele numbers in vesicular and nonvesicular samples was significant (Student *t* test, *P* < .001) as it was between vesicular and encephalitis samples (Student *t* test *P* < .05).

**Figure 4.**
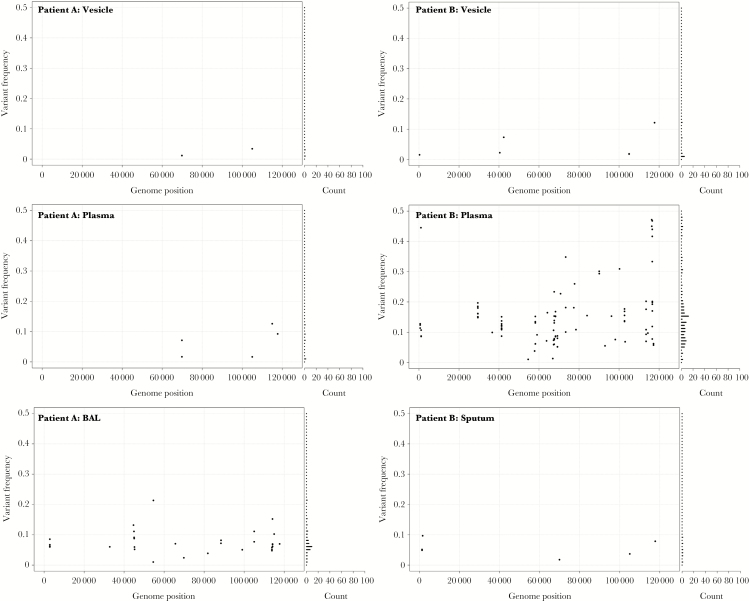
The genome-wide distribution and frequency scatter plots for all variant alleles in multiple samples from patients A and B. The x axis denotes genome position and y axis denotes the frequency of each variant allele (black circle). The number of variant alleles are binned at 1% frequencies and are displayed in a horizontal bar chart on the right side. Abbreviation: BAL, bronchoalveolar lavage.

**Figure 5.**
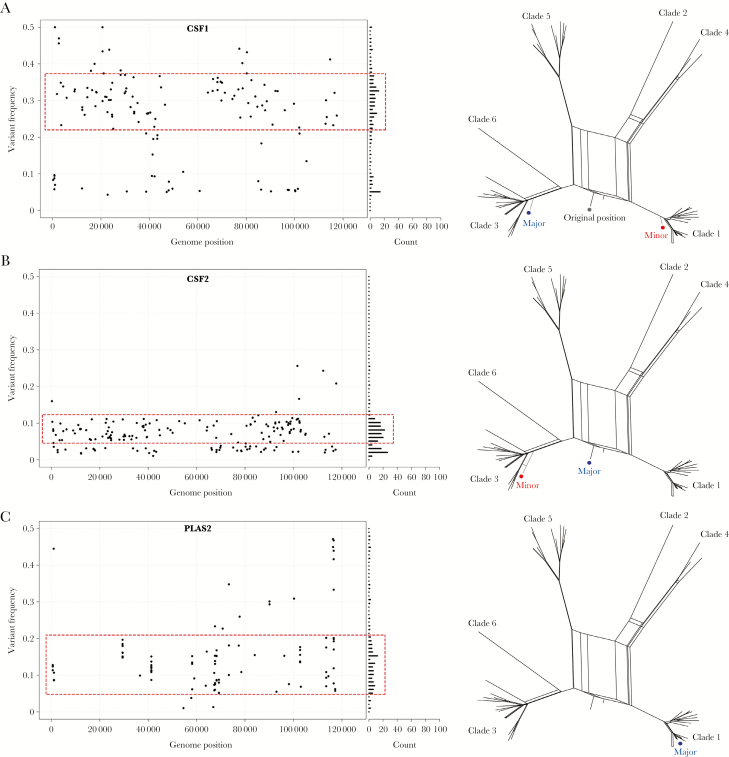
Evidence of varicella zoster virus superinfections in cerebrospinal fluid and plasma. *A–C*, The genome-wide distribution and frequency of all variant alleles shown as a scatter plot (left). X axis denotes genome position and y axis denotes the frequency of each variant allele (black circle). The number of variant alleles are binned at 1% frequencies and are displayed in a horizontal bar chart (right side). A normally distributed subpopulation of alleles (dotted red box) is suggestive of a mixed infection. Subpopulations are shown in the network phylogenies (right) (major sequence, blue circle; minor sequence, red circle). Abbreviations: CSF, cerebrospinal fluid; PLAS, plasma.

Nonvesicular samples also showed a different distribution of variant allele frequencies (Kolmogorov-Smirnov, *P* < .001) compared to vesicular fluid (Supplementary Figure 4). The 3 most variable samples (PLAS2, CSF1, and CSF2; Figure 2), had distinct patterns of variant allele frequencies suggestive of the presence of minor viral populations (Figure 5). Variant numbers sampled from multiple sites mostly reflected sample origin, with vesicular samples having lower numbers than samples taken from CSF, BAL, or plasma in the same patients (Figure 3 and 4). There was no difference in the number of variant alleles in vesicle samples by disease (Student *t* test, *P* = .50, varicella versus zoster) or immune status (Student *t* test, *P* = .79, immunocompetent versus immunocompromised). All bar 1 of the CSFs were from immunocompetent patients either with CNS disease alone or with concomitant herpes zoster and there were too few of the other sample types for statistical analysis.

### High Nucleotide Diversity Values are Indicative of Superinfections and/or Prolonged Infections

To investigate whether the increased nucleotide diversities of samples CSF1, CSF2, and PLAS2 were due to mixed infections, we substituted variant alleles within distinct frequency ranges into the consensus sequences for the same samples and generated additional network phylogenies. For CSF1, the putative minority genome was present at a frequency of 22%–46%. Substituting the variant alleles occurring at this frequency into the clade 3 consensus sequence resulted in a sequence that mapped most closely to clade 1 viruses and reexamining the CSF1 sequence data shows it not to be an interclade recombinant but rather a mixture of clades 1 (minority) and 3 (majority) viruses (Figure 5A). For CSF2, a normally distributed subpopulation was observed in a frequency range 6%–12%. The minority sequence was found to cluster most closely with clade 3 viruses (Figure 5B). The low-level clade 3 variant was distinct from the dominant consensus sequence, a recombinant clade 1/3 virus (Figure 5B) [9]. PLAS2, where the putative subpopulation, albeit less distinct than for CSF1 and CSF2, was distributed between frequencies of 6% and 22%, and did not yield a different clade type. The distribution of variant alleles across the genome also differed for PLAS2 with several groups of variant alleles appearing in localized clusters, not observed for other samples, including CSF1 and CSF2 (Figure 5C). Further analysis of PLAS2 showed over 39% of variant alleles clustered in 3 glycoproteins, ORF31 (glycoprotein B), ORF37 (glycoprotein H), and ORF68 (glycoprotein E), as well as the ORF20 (capsid) and ORF22 (large tegument) genes. Inspection of sequence read quality scores and alignments strongly suggested these variant alleles did not arise through artefacts, although insufficient material remained for polymerase chain reaction or resequencing. As a control, we repeated the analyses for all other samples with 10 or more variant alleles (Table 1 and Supplementary Figures 1 and 2) and determined that these infections had neither mixed clade infections nor mutations clustering in the above open reading frames.

## DISCUSSION

These results are compatible with previous conclusions that individual skin vesicles are typically founded by 1 to 3 virions [13], a significant population bottleneck that limits subsequent viral population diversity within vesicles. Our previous studies made use of the vOka vaccine, which contains a mixture of related strains each of which carries a different subset of the approximately 140 known vaccine mutations [15]. Each vOka virion is effectively “barcoded,” enabling tracking of their fate following vaccine inoculation [6, 15, 16]. In this study, 24 out of 27 sampled vesicle populations contained fewer than 10 variant alleles. High and low copy number vesicles occurred within the same patient (eg, D, VES19 and VES25 and F, VES6 and VES25) and differences in vesicular copy number were not explained by clinical characteristics (age, immunosuppression, disease) (Table 1). We therefore suggest that the 3 vesicular fluids (VES 6, VES9, and VES19) with higher diversity (Figure 3) are explained by these samples containing genetic material inadvertently sampled from multiple vesicles; similar levels of diversity were obtained when pooling deep sequence from all 5 patient D vesicles (Supplemental Figure 3).

By contrast, although numbers were small, we observed significantly higher diversity in CSF from cases of encephalitis, with 7 out of 8 CSF samples from these patients having greater than the median numbers of variants, as compared with vesicular fluid and CSF samples from patients with meningitis (CSFs 8–11) (*P* < .05). Viral diversity was not related to viral load, which we have previously shown can be inferred in relatively acellular fluids such as CSF from the percentage of on-target reads [14]. Instead the highest diversity in CSF1 and CSF2 was due to mixed infection with 2 or more different VZV clades. CSFs 1 and 2 came from immunocompetent patients presenting with acute encephalitis and no skin rash. Both patients had past histories of chickenpox with no history of recent exposure. We conclude that their encephalitides are likely to have arisen from viral reactivation, most probably, given the mixed genotypes present, originating from multiple neurons or ganglia. We have previously shown latent infection and reactivation in the same individual, at different times, of VZV belonging to different clades [17]. How often infection and latency with multiple viruses occurs is not known, especially as distinguishing viruses from the same clade is difficult. Nonetheless, in an observational study of African and Asian patients who developed zoster after migrating as adults to the United Kingdom, having had varicella in their country of origin, 30% reactivated a UK genotype rather than the clades 4 and 5 prevalent in their countries of origin [18]. Reinfection and latency followed by reactivation of European clade wild-type viruses is also well described for subjects who have seroconverted to the Japanese vOka vaccine strain [19]. We and others have previously shown that the natural history of VZV is characterized by extensive genetic recombination between VZV strains from different geographic clades [11, 20]. Recombination requires 2 distinct virions to be present in the same cell. Our finding of 2 patients with evidence for multiple strains replicating simultaneously in CSF highlights the nervous system as a potential site for recombination to occur.

Other encephalitis samples such as CSF7, with high variant numbers at low frequencies, could represent reactivation of the same clade-virus from multiple sites, giving a similar allelic frequency profile as the pooled patient D vesicle alleles (Supplementary Figure 3). In contrast, virus from cases of meningitis shows similar diversity to that of vesicular fluid, pointing to the possibility that self-limiting VZV meningitis is caused by reactivation and replication of VZV from very few neurons. Alternatively, the diversity observed in encephalitis cases could be due to accumulating mutations, perhaps driven by the immune system. In this scenario, more-severe cases where virus spreads to brain parenchyma to cause encephalitis could be due to poorer immune control with more evidence of escape mutations. This latter hypothesis would not, however, account for the mixed clades observed in CSFs 1 and 2, and is less easy to explain in the context of young immunocompetent patients such as those described here.

Two other samples with higher viral diversity, PLAS2 and BAL1, both came from patients with profound immunosuppression. We have previously shown the presence of a mixed vOka founder population in endotracheal aspirates from a case of vOka pneumonitis in an immunocompromised child, presumably from the multiple foci of infection in the lungs. Vesicular fluid from the same child was, as expected, derived from a single virion [21]. Extrapolating to this study, the larger numbers of variant alleles present in bronchoalveolar lavage and plasma samples could have arisen from multiple virions spilling over from the tissues in which they were replicating. Prolonged viral replication in the presence of poor immune control is likely to exacerbate diversity and may account for the extremely high numbers of variants seen in PLAS2 from patient B, a renal transplant recipient with an undiagnosed, disseminated VZV infection for at least 10 days prior to the sample being collected [22]. The high concentration of these variants in surface-expressed proteins may reflect viral mutagenesis in response to immune pressures. Patient B developed respiratory failure as a late manifestation of their illness [22] and a sputum sample taken at the same time as PLAS2 showed low diversity with few variant alleles. By contrast Patient A, also a renal transplant recipient, had very few variant alleles in plasma (PLAS1) although more in the BAL (BAL1). Patient A presented with varicella and pneumonitis, and the plasma sample was taken at presentation approximately 19 days following contact with patient B, the source of patient A’s infection (ie, at the onset of illness and possibly before extensive replication had occurred). The higher nucleotide diversity of VZV populations in the lungs, compared to plasma, could be explained by virus having spread more widely and replicated for longer in the lungs at this early stage. Plasma samples from later in the course of infection were not available but we would speculate that they may have shown increasing diversity. Patient A did not show the variant clustering seen in patient B. Because patient A, unlike patient B, was VZV-naive before infection [22], their lack of viral heterogeneity may reflect the absence of immune-driven mutagenesis.

In summary, using genetic studies we have shown that, like the VZV vOka vaccine strain, vesicles arising during VZV wild-type infections are likely to be founded by few virions, a major bottleneck event that results in restricted genome diversity within vesicles as the founder VZV population expands [13]. By contrast, we found evidence that cases of encephalitis may be more diverse. The evidence of mixed clade infections and high viral diversity are most parsimoniously explained by reactivation of multiple founder viruses causing encephalitis with reactivation of a few causing the less diverse meningitis. Alternatively, we cannot exclude the possibility that some of the increased diversity seen in encephalitis (although not the mixed clades) is due to accumulating escape mutations, possibly resulting from incomplete immune control. Fifty percent of VZV encephalitis cases occur in association with immunosuppressive conditions in which viral reactivation is known to be more common. The factors predisposing to widespread VZV reactivation in younger immunocompetent patients are more difficult to explain. It is possible that in these subjects genetic factors are important. This hypothesis has resonances with the finding that herpes zoster in younger patients (<40 years) confers a significantly higher lifetime risk of stroke, a finding that has been mooted [23] to reflect a genetic predisposition to VZV reactivation both symptomatic and asymptomatic. Importantly, although sample numbers were low we do not find any evidence that VZV strains recovered from the CNS share polymorphisms that distinguish them from VZV strains recovered from other (non-CNS) compartments. These findings suggest that VZV infection of the CNS is unlikely to be due to single viral polymorphisms.

## Supplementary Data

Supplementary materials are available at *The Journal of Infectious Diseases* online. Consisting of data provided by the authors to benefit the reader, the posted materials are not copyedited and are the sole responsibility of the authors, so questions or comments should be addressed to the corresponding author.

Figure S1

Figure S2

Figure S3

Figure S4

Supplementary Figures and Legends

Supplementary Tables

## Notes

***Acknowledgments.*** The authors would like to thank Kate Cawthorn and Pamela Molyneux for assistance in obtaining samples used in this study. We acknowledge support from the Medical Research Council (MRC) and Biomedical Research Centre (BRC) for the University College London/ University College London Hospitals (UCL/UCLH) Pathogen Sequencing Pipeline as well as the UCL Legion High Performance Computing Facility, and associated support services, in the completion of this work. All research at Great Ormond Street Hospital NHS Foundation Trust is made possible by the National Institute for Health Research (NIHR) Great Ormond Street Hospital Biomedical Research Centre.

***Disclaimer***. The views expressed are those of the authors and not necessarily those of the NHS, the NIHR, or the Department of Health.

***Financial support.*** This work was supported by a UK MRC New Investigator Award to D. P. D; UCL/UCLH BRC (J. B.); Action Medical Research (grant number GN2424 to C. J. H); Swedish Research Council (P. N. and T. B.). The work was also support by an NIHR Fellowship (grant number DRF-2013-06-168 to F. M.), the Meningitis Research Foundation (grant number 0904.0), an NIHR Programme Grant in Applied Research (grant number RP-PG-0108-10048 to T. S.), and the NIHR Health Protection Research Unit in Emerging and Zoonotic Infections, University of Liverpool.

***Potential conflicts of interest.*** All authors: No reported conflicts of interest. All authors have submitted the ICMJE Form for Disclosure of Potential Conflicts of Interest. Conflicts that the editors consider relevant to the content of the manuscript have been disclosed.
