# Supplementary figures and images for: High Viral Diversity and Mixed Infections in Cerebral Spinal Fluid From Cases of Varicella Zoster Virus Encephalitis
Source: J Infect Dis. 2018 Jul 7;218(10):1592–601. doi: 10.1093/infdis/jiy358 (PMC6173578; doi:10.1093/infdis/jiy358)

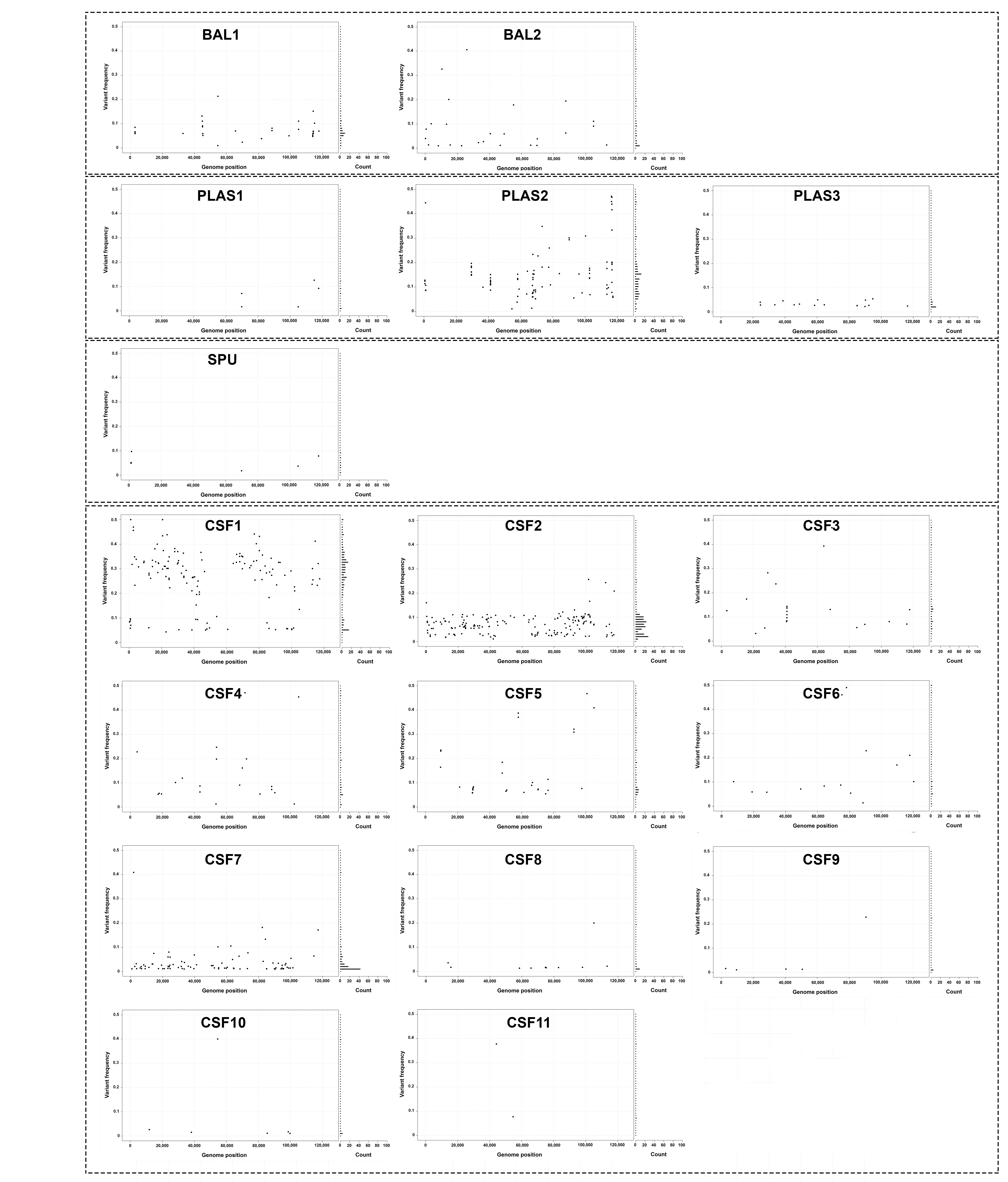

Supplement: Figure S1 [file jiy358_suppl_figure-s1.png]

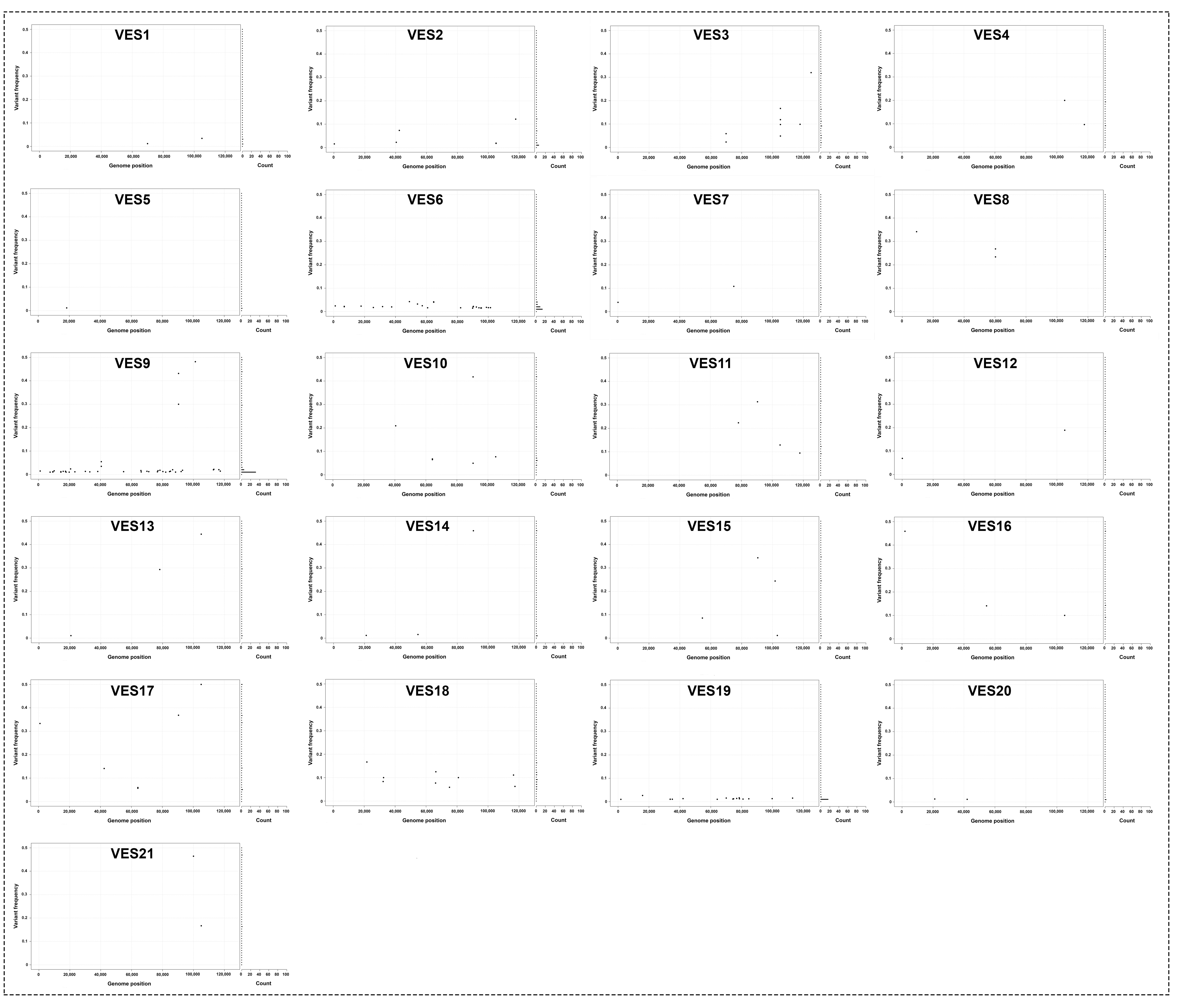

Supplement: Figure S2 [file jiy358_suppl_figure-s2.png]

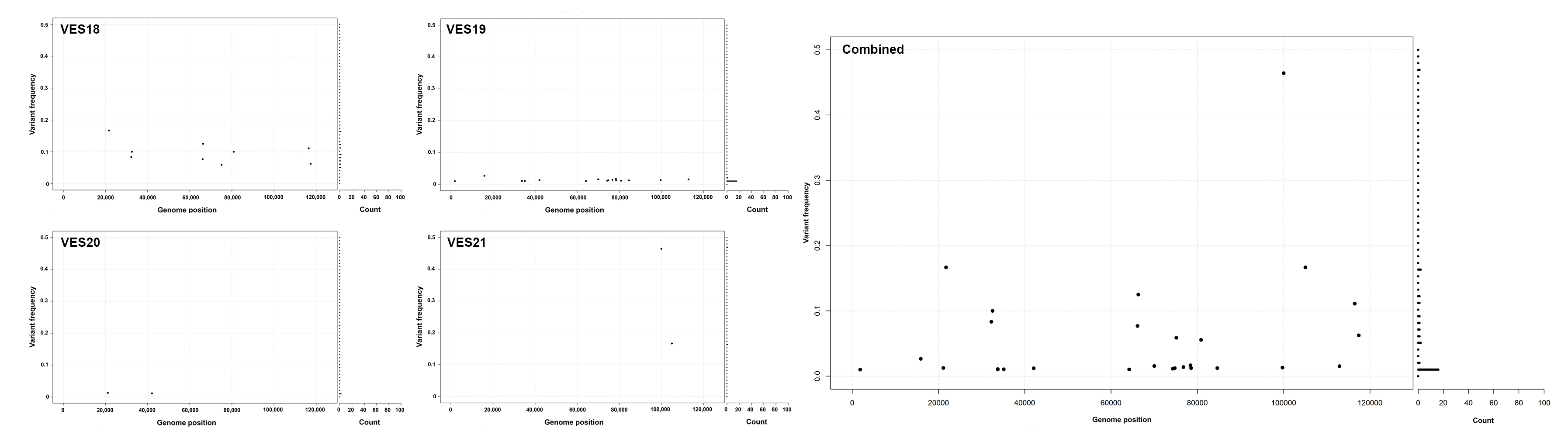

Supplement: Figure S3 [file jiy358_suppl_figure-s3.jpeg]

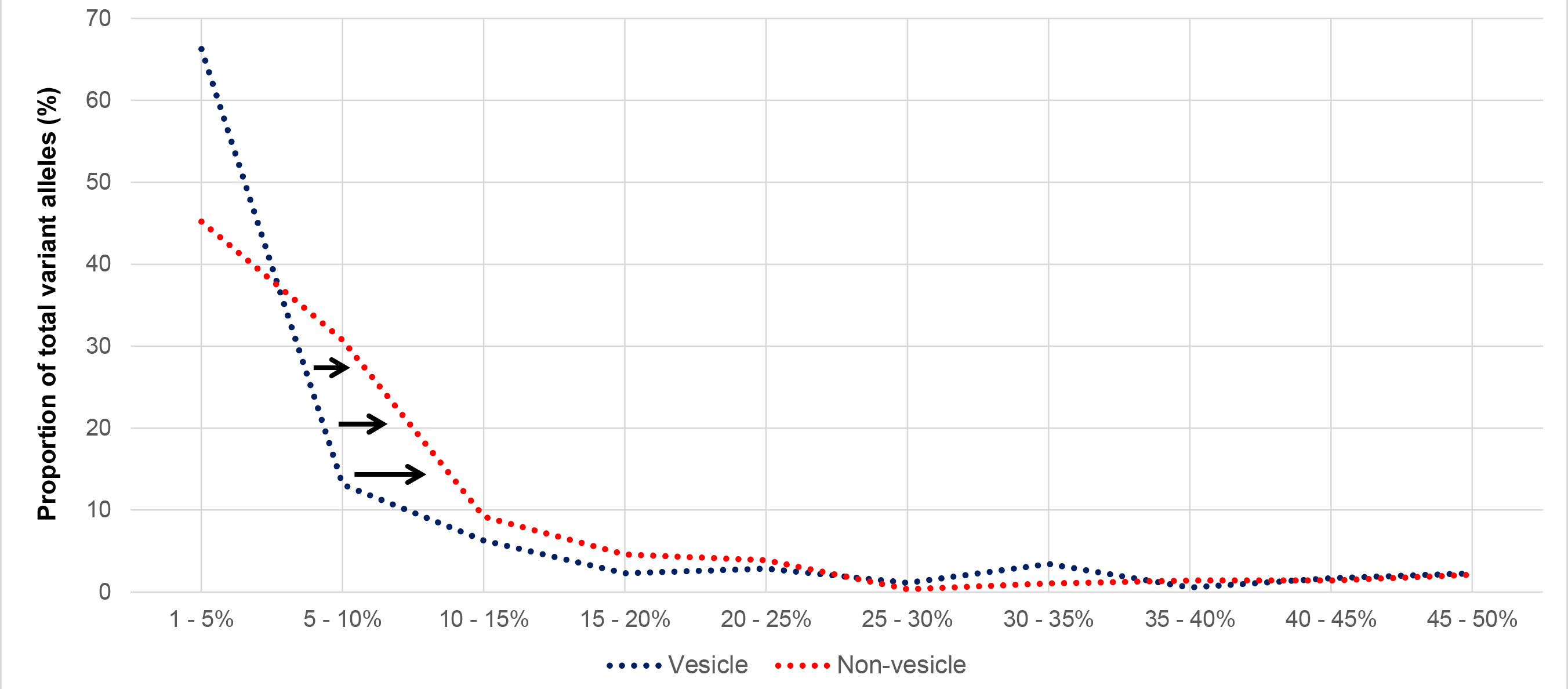

Supplement: Figure S4 [file jiy358_suppl_figure-s4.png]
