# Supplementary material for: High Viral Diversity and Mixed Infections in Cerebral Spinal Fluid From Cases of Varicella Zoster Virus Encephalitis
Source: J Infect Dis. 2018 Jul 7;218(10):1592–601. doi: 10.1093/infdis/jiy358 (PMC6173578; doi:10.1093/infdis/jiy358)
Supplement: Supplementary Figures and Legends [file jiy358_suppl_supplementary_figures-legends.docx]

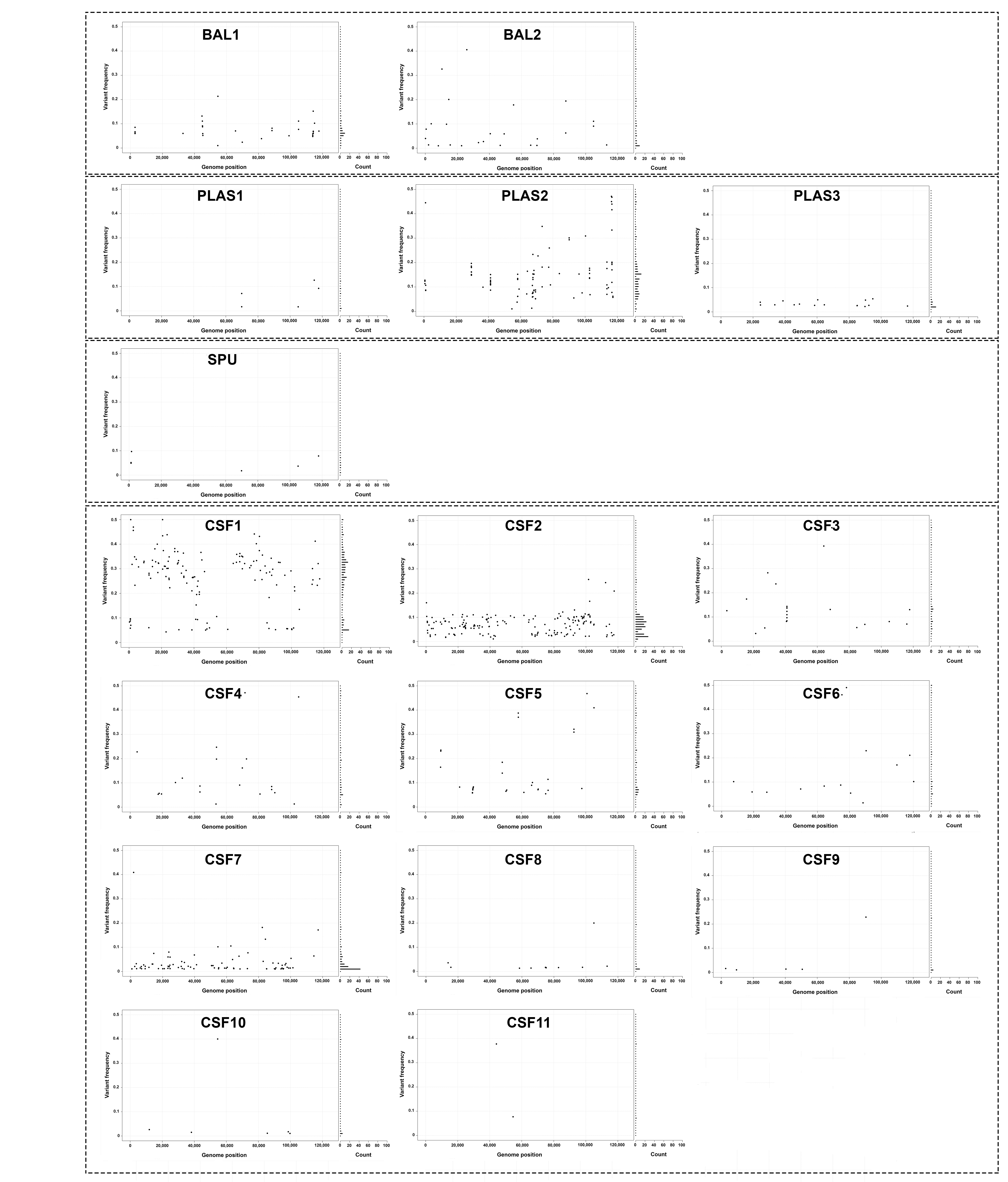
**Supplementary Figure 1: Intra-sample VZV populations in plasma, sputum, CSF, and BAL samples.**

The genome-wide distribution and frequency of all variant alleles shown as a scatter plot for each VZV population within individual plasma, sputum, CSF, and BAL samples used in this study. X-axis denotes genome position while y-axis denotes the frequency of each variant allele (black circle). A frequency histogram is laid along the right-hand side of the plot. Here, counts denotes the number of variant alleles within a given frequency range (e.g. 0-1%, 1-2%, 2-3% etc).


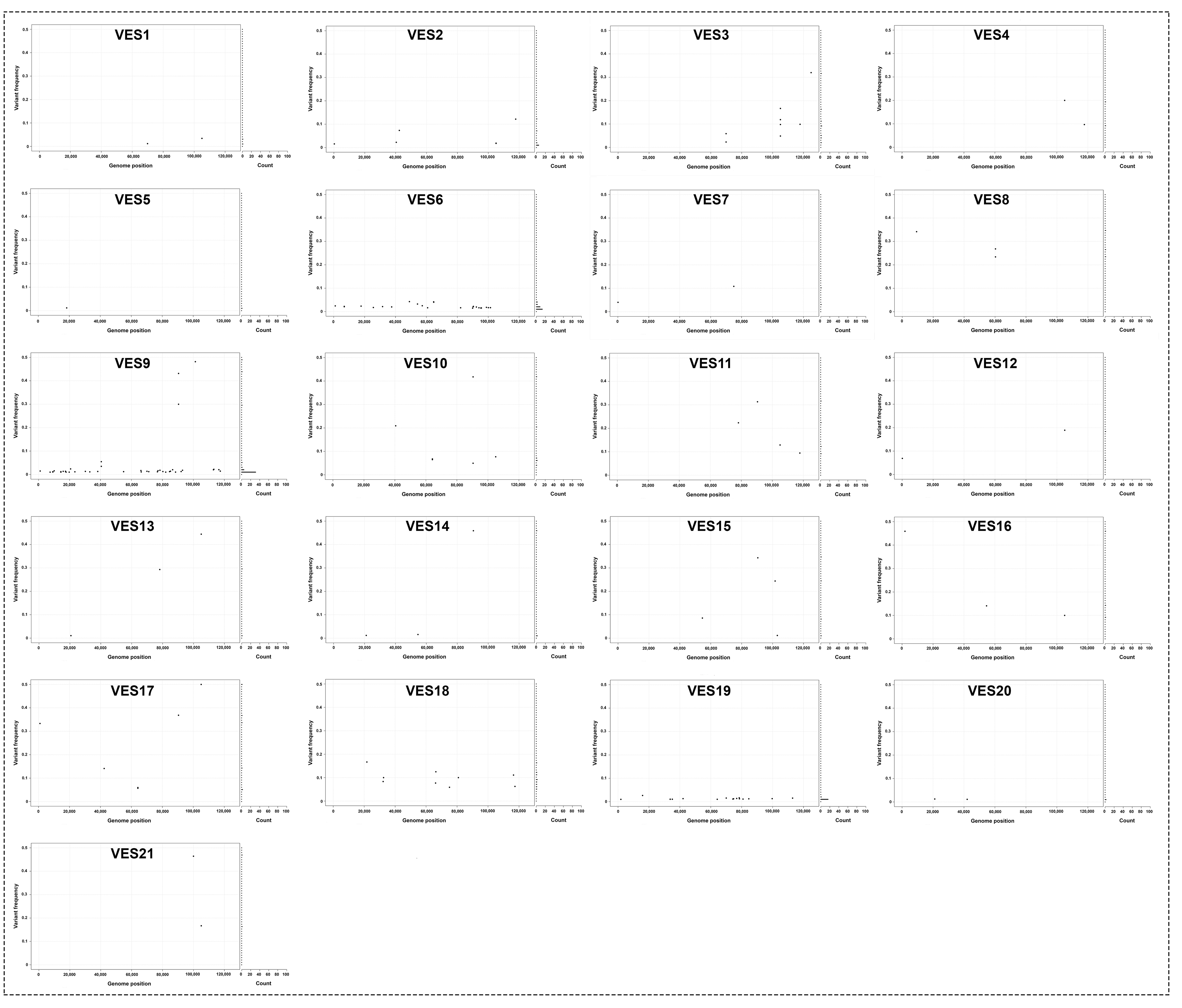


**Supplementary Figure 2: Intra-sample VZV populations in vesicle samples.**

The genome-wide distribution and frequency of all variant alleles shown as a scatter plot for each VZV population within individual vesicle samples used in this study. X-axis denotes genome position while y-axis denotes the frequency of each variant allele (black circle). A frequency histogram is laid along the right-hand side of the plot. Here, counts denotes the number of variant alleles within a given frequency range (e.g. 1-2%, 2-3% etc). Note, samples with no variant alleles above 0% frequency are not shown (i.e. CSF12).


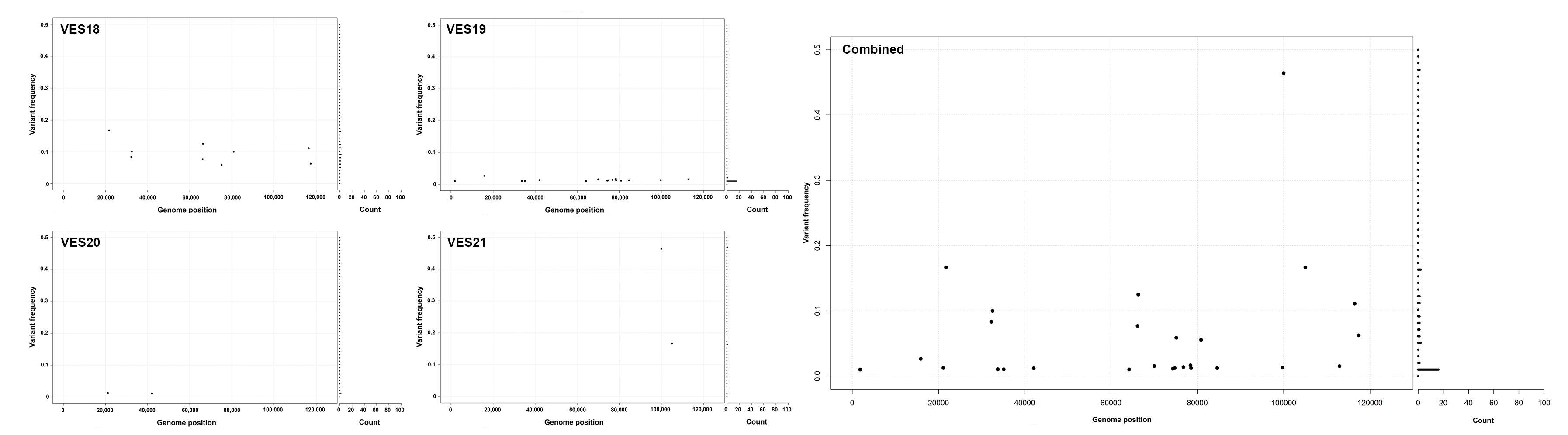
**Supplementary Figure 3: Intra-samples VZV populations (separate and combined) in vesicle samples from patient D**

The genome-wide distribution and frequency of all variant alleles shown as a scatter plot for each VZV population within four of five vesicles sampled from Patient D (each shown as a separate panel). The combined population of all four vesicle samples is shown in the right panel. X-axis denotes genome position while y-axis denotes the frequency of each variant allele (black circle). A frequency histogram is laid along the right-hand side of the plot. Here, counts denotes the number of variant alleles within a given frequency range (e.g. 0-1%, 1-2%, 2-3% etc). Note, samples with no variant alleles above 0% frequency are not shown (i.e. VES22-VES27).


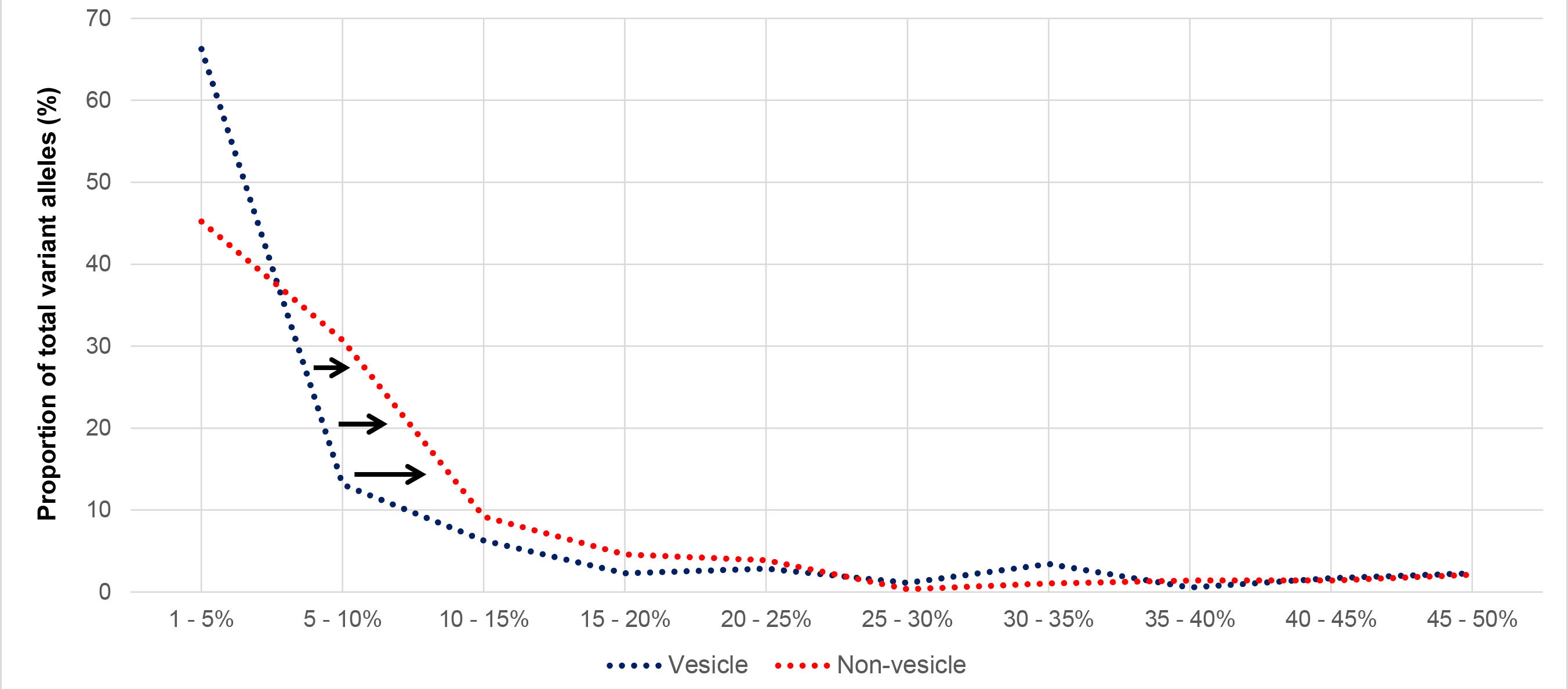


**Supplementary Figure 4: VZV variant allele frequency distributions in vesicle and non-vesicle populations.**

Total variant allele counts were generated for vesicle and non-vesicle populations (excluding CSF1, CSF2 and PLAS2) and proportionally binned at 5% intervals. E.g. for vesicle populations, ~68% of variant alleles are at frequencies of 1-5% compared to ~45% for non-vesicle populations. A marked shift in the distributions is indicated by black arrows, representative of a shift in variant frequency allele distribution.
