# Supplementary material for: High Viral Diversity and Mixed Infections in Cerebral Spinal Fluid From Cases of Varicella Zoster Virus Encephalitis
Source: J Infect Dis. 2018 Jul 7;218(10):1592–601. doi: 10.1093/infdis/jiy358 (PMC6173578; doi:10.1093/infdis/jiy358)
Supplement: Supplementary Tables [file jiy358_suppl_supplementary_tables.pdf]

VZV isolate / reference strain	Genbank ID
Dumas	NC_001348
HJ0	AJ871403
SD	DQ479953
SVETA	EU154348
BC	AY548171
M2DR	DQ452050
Var160	KC112914
Bandim10	KM355704
Bandim6	KM355701
Bandim21	KM355715
Var/Cli/UK/Ves/2403/2013	KP771913
Var/Cli/UK/BAL/2403/2013	
Var/Cli/UK/Plasma/2403/2013	
Var/Cli/UK/Ves/0803/2013	KX262864
Var/Cli/UK/Sputum/0603/2013	
Var/Cli/UK/Plasma/0603/2013	
Var/Cli/UK/CSF/2912/2012	KP771908
Var/Cli/UK/Ves/0201/2013	
Var/Cli/Ves/UK/ZC/2017	MG764313
Var/Cli/Ves/UK/3323/2015	MG764305
Var/Cli/Ves/UK/9394/2015	MG764308
Var/Cli/Ves/UK/321/2015	MG764307
Var/Cli/BAL/UK/2402/2009	KP771904
Cli/UK/CSF/3009/2011	KP771890
Cli/UK/CSF/2909/2011	KP771889
KV6-2313	KP771901
KV6-3127	KP771902
KV8-1390	KP771903
Zos/Cli/CSF/Sing/1008/2008	KP771922
Men/Cli/CSF/UK/UKM1018/2017	MG764309
Men/Cli/CSF/UK/UKM1211/2017	MG764310
Men/Cli/CSF/UK/UKM0338/2017	MG764311
Men/Cli/CSF/UK/UKM0624/2017	MG764312
Var/Cli/UK/CSF/0102/2013	KP771907
Var/Cli/UK/Ves/2203/2013	KP771912
Var/Cli/Ves/UK/724/2015	MG764306
DE10-567	KP771899
DE10-581	KP771900
DE10-1515	KP771891
DE10-2660	KP771893
DE10-2704	KP771894
DE10-3378	KP771895
DE10-4367	KP771896
DE10-4582	KP771897
DE10-5454	KP771898
Var/Cli/Ves/Sing/1308/2008	KP771919
Var/Cli/Ves/Ger/31/2005	KP771915
Var/Cli/Ves/Ita/51/2006	KP771918
DE10-2480	KP771892

Table S1: VZV isolates and reference strains used in this study for network phylogeny

Sample ID	% genome coverage at depth X			Mean read depth per base
	1X	20X	100X	
BAL2	99.75	97.78	92.86	971
VES3	100.00	99.82	98.52	1519
PLAS2	99.62	97.33	75.37	275
VES2	100.00	99.45	98.19	822
SPU	100.00	99.00	97.72	1131
PLAS1	99.99	99.35	98.13	1119
VES1	99.99	99.05	97.82	1216
BAL1	99.99	98.70	94.56	790
CSF3	99.83	98.57	96.66	987
VES27	99.97	98.98	97.70	972
CSF12	99.99	98.95	97.78	980
VES26	99.97	99.69	98.60	1298
VES4	99.97	99.55	98.12	542
VES5	99.78	99.03	97.12	337
PLAS3	99.52	92.97	0.00	34
VES6	99.52	96.79	0.21	50
VES25	99.94	99.65	98.51	1322
VES9	99.87	98.33	91.78	782
VES24	99.98	99.71	98.65	1325
VES10	100.00	99.84	98.95	1851
VES11	99.99	99.47	98.37	1327
VES12	99.99	99.62	98.62	1099
VES13	99.96	99.48	98.31	1372
VES14	99.85	99.16	96.46	549
VES15	99.97	99.09	97.51	894
VES7	99.87	98.59	91.32	534
VES8	99.85	98.12	83.39	213
CSF4	100.00	99.81	98.57	596
CSF5	100.00	99.99	99.80	5206
CSF6	99.99	99.88	99.41	3889
CSF2	99.95	98.68	86.04	135
CSF1	99.96	98.73	97.74	888
CSF7	99.84	98.21	90.35	285
VES16	99.93	99.16	97.67	892
VES17	99.99	99.79	98.99	1500
VES23	100.00	99.72	98.54	1018
CSF8	99.85	97.51	21.30	78
CSF9	99.83	99.24	96.85	324
CSF10	99.62	96.75	1.06	54
CSF11	99.84	99.12	95.94	213
VES20	99.88	98.23	71.31	110
VES21	99.86	98.97	97.53	492
VES18	98.46	0.34	0.00	8
VES22	99.93	99.14	97.74	544
VES19	99.77	97.61	6.24	77

Table S2: Coverage metrics
